# Percutaneous Ethanol Injection in Combination with Laser Ablation for a 100 ml Partially Cystic Thyroid Nodule

**DOI:** 10.1155/2018/8046378

**Published:** 2018-02-15

**Authors:** Roberto Negro, Gabriele Greco

**Affiliations:** Division of Endocrinology, “V. Fazzi” Hospital, Lecce, Italy

## Abstract

Until nonsurgical techniques like laser ablation (LA) or radiofrequency became available, patients suffering from large nodules with compressive symptoms were addressed to surgery. We describe the case of a 59-year-old woman with a large, partially cystic thyroid nodule having a volume of about 100 ml. As the patient refused surgery, despite her constant local discomfort, such large partially cystic nodule underwent several percutaneous ethanol injections (PEI) and then was submitted to LA. The combination of these two procedures allowed firstly complete disappearance of the cystic component and secondly a significant reduction of thyroid nodule, which finally measured 17 ml in volume (82% reduction compared to baseline). This case demonstrates that even in very large partially cystic nodules LA preceded by PEI represents a valid alternative to surgery.

## 1. Introduction

Until few years ago, surgery was the only option for some either malignant or benign thyroid diseases. Surgery may be indicated for thyroid cancer or nodules suspected for malignancy, for multinodular toxic or nontoxic goiter with compressive symptoms, and for Graves' disease especially when radioactive treatment is refused by the patient or is contraindicated. Most thyroid nodules are benign and remain asymptomatic, but some progressively grow and may cause compressive symptoms. Surgery is the standard therapeutic approach for thyroid lesions that, even if benign at fine-needle aspiration (FNA), are steadily growing over time [1]. However, surgery is expensive, necessitates life-long thyroid hormone replacement therapy, and may be followed, although infrequently, by permanent complications [2]. Over the last two decades nonsurgical, minimally invasive techniques have been proposed for the treatment of benign thyroid nodules when surgery is contraindicated or declined [3].

## 2. Case Presentation

We report the case of a 59-year-old lady who was referred to our thyroid clinic for a known nodule of the right lobe, which provoked a progressive difficulty in swallowing and breathing. The patient said that she was euthyroid, was diagnosed with a large thyroid cystic nodule of the right lobe since a long time, and underwent periodic fluid drainage of the cyst in another hospital and that the cyst regularly returned back to the original size. The patient always refused surgery.

**Figure 1:**
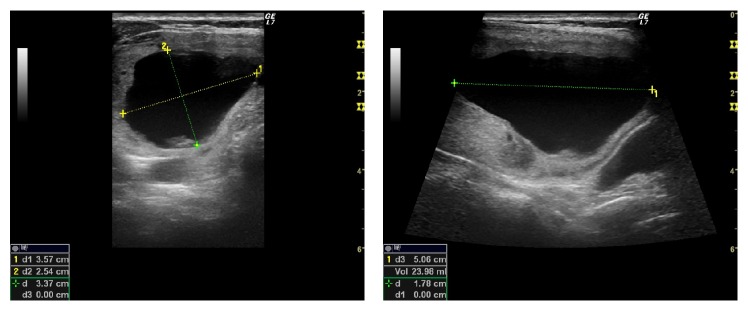
Right lobe thyroid cystic nodule at baseline.

Our first ultrasound examination in January 2016 revealed a thyroid nodule of the right lobe that measured 5.9 × 3.8 × 8.0 cm (volume 94.75 ml); the nodule had a cystic component that measured 3.6 × 2.5 × 5.0 cm (volume 24 ml) (Figure 1); the isthmus and left lobe were normal.

**Figure 2:**
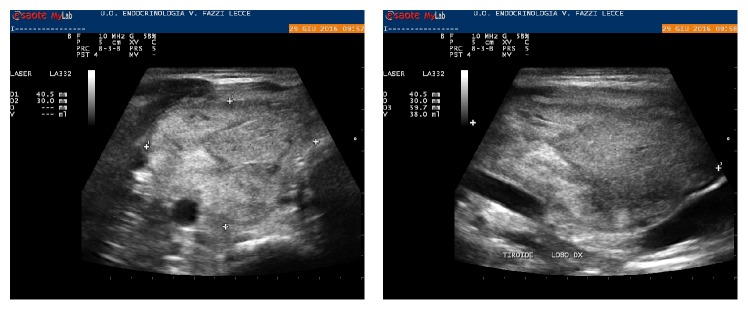
Right lobe thyroid nodule after six percutaneous ethanol injections.

Thyroid function tests were as follows: TSH, 1.79 mIU/L (0.3–3.6); FT4, 1.0 ng/dl (0.8–1.7); thyroglobulin, 305.8 ng/ml (0.2–70); TPOAb and TgAb, negative; and calcitonin, <1.0 pcg/ml (<10). FNA of the peripheral portion of the nodule was obtained with a negative cytologic result. From January to June 2016, the patient underwent 6 drainages of the cystic fluid (about once a month) with percutaneous ethanol injection (PEI) that obtained complete disappearance of the liquid component. At this time, a spongiform nodule measuring 4.0 × 3.0 × 6.0 cm (volume: 38 ml) was documented, with a 60% reduction with respect to baseline (Figure 2). At the end of June, the patient underwent laser ablation (LA) of the thyroid nodule. In a single session, two fibers were placed within the nodule, and, following the pull-back technique, 15,000 joules were delivered [4].

**Figure 3:**
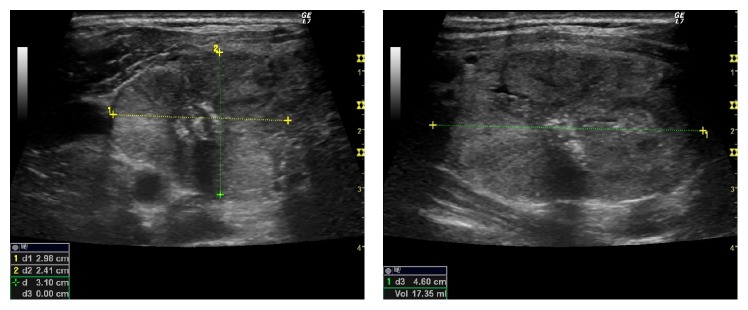
Right lobe thyroid nodule six months after laser ablation.

Six months after LA, the nodule measured 3.0 × 2.4 × 4.6 cm (volume: 17 ml, 82% reduction compared to baseline) (Figure 3); the patient was completely asymptomatic and was still euthyroid.

The above-mentioned clinical case represented the largest cystic nodule we have treated with PEI + LA. The use of LA in thyroid cyst is not a novelty. Døssing et al. demonstrated in a prospective randomized trial that aspiration of the cystic fluid followed by LA obtained a successful outcome (cyst volume ≤ 1 ml) in 68% of patients versus 18% of cases when aspiration was not followed by LA [5]. The largest cystic nodule treated in this study measured 26.8 ml, less than one-third of the one treated in our patient. The combined use of PEI followed by LA allowed firstly removing the liquid component and secondly shrinking the volume of the solid nodule. LA has been successfully used to reduce the volume of thyroid nodules and has been proven to be superior to levothyroxine suppressive treatment; then, for several years, many patients, especially those suffering from spongiform nodules, benefited from LA and were spared to surgery [6–8]. Moreover, an unexpected chance of success of LA has also been demonstrated when used in combination with radioactive therapy for large single and multiple autonomous functioning nodules [9, 10].

The successful outcome of our patient demonstrates that the use of LA in combination with other techniques (PEI in this case) opens new perspectives in the treatment of thyroid disease, which were exclusively prerogative of surgical treatment. Perhaps it is premature to consider such nonsurgical treatments as standard of care, but for sure they can be utilized by skilled physicians for many cases in which until few years ago the surgical choice was the only option.

The short follow-up time (6 months) represents a major limitation in this case report. In this view, some points may be considered: LA usually obtains the greatest effect in volume reduction within 6 months; the volume reduction has been demonstrated to be stable over the time; when necessary, the residual nodule may be submitted to further LA sessions to obtain even more significant shrinkage [6, 8]. On the other hand, it should be noted that studies demonstrating persistent nodules reduction for a very long time and in a sizeable sample of patients are lacking.
